# Caecal perforation from CT colonography: an unexpected complication

**DOI:** 10.1093/jscr/rjad086

**Published:** 2023-03-04

**Authors:** Vijay Pather, Justin Rodrigues, Tasmea Sefa, Ewan MacDermid

**Affiliations:** Department of General Surgery, Nepean Hospital, Kingswood, NSW, Australia; Department of General Surgery, Nepean Hospital, Kingswood, NSW, Australia; Department of General Surgery, Nepean Hospital, Kingswood, NSW, Australia; Department of General Surgery, Nepean Hospital, Kingswood, NSW, Australia; Department of Colorectal Surgery, Nepean Hospital, Kingswood, NSW, Australia

## Abstract

Computer tomography colonography (CTC) is an increasingly utilized diagnostic modality in Australia. CTC aims to image the entire colon, and is often used in higher risk patient populations. Colonic perforation following CTC is a rare complication, with only 0.008% of patients undergoing CTC requiring surgical intervention. Most published cases of perforation following CTC are due to identifiable causes, often involving the left colon or rectum. We present a rare case of caecal perforation following CTC, requiring right hemicolectomy. This report highlights the need for high suspicion for CTC complications, despite their rarity, and the utility of diagnostic laparoscopy for diagnosis in atypical presentations.

## INTRODUCTION

Computer tomography colonography (CTC) is an increasingly accessible diagnostic modality used often in a higher risk patient population. CTC is performed by inflating the colon with carbon dioxide after the insertion of a flexible rectal catheter. Similar to traditional colonoscopy, it is essential that patients have a bowel-prepped colon. After gas insufflation, CTC uses computer tomography to create a 3D image of the colon to aid in the detection of colonic polyps [1]. Compared with colonoscopy, CTC is less invasive and is therefore associated with lower rates of complications such as bleeding, perforation and anaesthetic complications [2]. A meta-analysis has described the rate of colonic perforation from CTC as 0.04%, being as low as 0.02% in asymptomatic patients [3]. Despite this, there is a paucity in the literature specifically describing right-sided perforation. We present a rare case of caecal perforation following CTC, requiring right hemicolectomy.

## CASE REPORT

**Figure 1:**
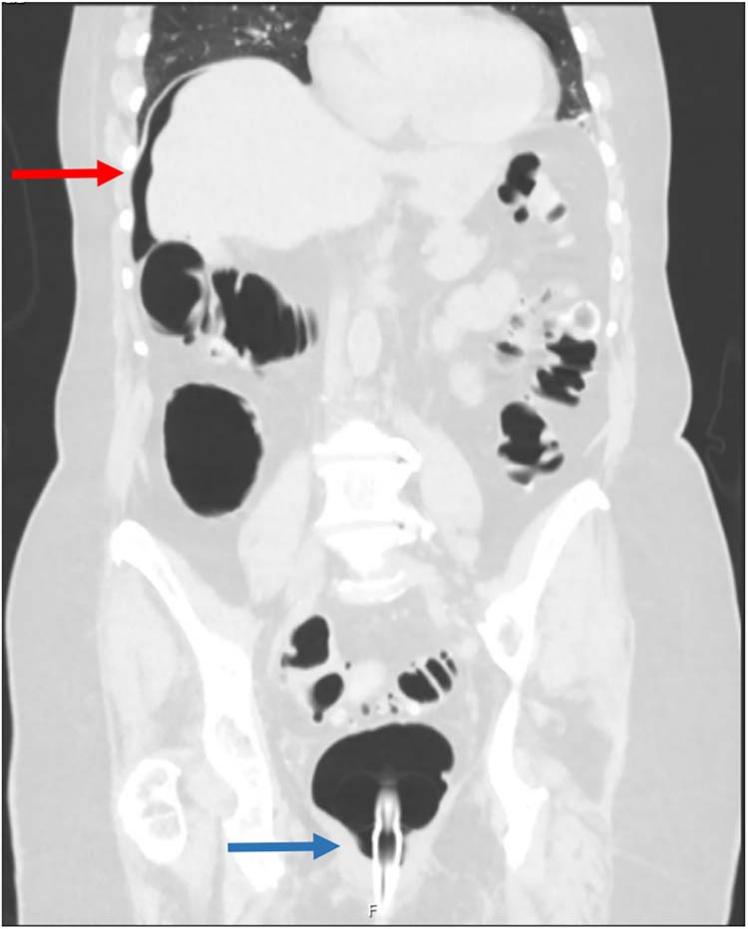
Coronal CT scan following gas insufflation; the red arrow points to pneumoperitoneum above the liver, and the blue arrow points to a rectal catheter.

**Figure 2:**
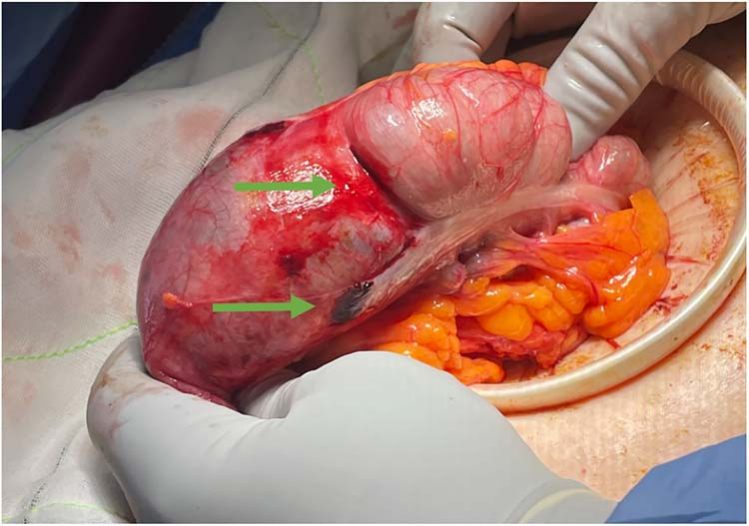
Clinical photograph of the caecum following laparoscopy; the green arrows point to a large tear in caecum and a tear overlying the taenia coli.

An 85-year-old female was referred from a peripheral hospital with large volume pneumoperitoneum detected during CTC (Fig. 1). CTC was performed using automated gas insufflation via a Foley catheter. Pneumoperitoneum was detected immediately after gas insufflation, therefore the CTC was abandoned. Despite the radiological findings, on review she was asymptomatic, without abdominal pain, tachycardia or fever. Her past medical history was significant for sigmoid diverticulitis, with no previous history of abdominal surgery. She underwent an incomplete colonoscopy 3 weeks prior, abandoned due to tortuous sigmoid colon anatomy. Despite being asymptomatic, she underwent diagnostic laparoscopy due to the large volume of pneumoperitoneum. At laparoscopy she was found to have a large caecal tear with two small areas of non-viable serosa (Fig. 2), and no gross intra-abdominal contamination. She proceeded to a laparoscopic right hemicolectomy with a subsequent uneventful post-operative recovery and benign histopathology.

## DISCUSSION

CTC is indicated for colonic screening, and is commonly performed due to incomplete or failed colonoscopy. In the event of neoplastic lesions which preclude caecal intubation with colonoscopy, CTC can be used for excluding synchronous lesions in the remaining colon [1]. CTC is preferred when colonoscopy is contraindicated due to patient factors, such as in frail or elderly patients [1]. Perforation rates from CTC are as low as 0.02% in asymptomatic patients, with only 0.008% of patients requiring surgical intervention [3]. Perforation from CTC can be due to both patient factors and insufflation technique, with an increased risk associated with manual air insufflation (88%) when compared with gas [2]. While it is unclear if the type of rectal catheter used correlates to perforation, it is hypothesized that flexible Foley catheters are safer than rigid rectal tubes [2]. Patient factors such as age, corticosteroid use, abdominal wall herniation and connective tissue disorders have been associated with an increased risk of perforation, and thus are considered contraindications to CTC [4]. Bowel pathology such as colonic obstruction, colitis or diverticulitis 6 weeks prior to CTC, and deep colonic biopsy prior to CTC are also associated with an increased risk of perforation [2]. Colonoscopy is preferred over CTC in high-risk patient populations. This includes patients with long-standing inflammatory bowel disease, or in patients with hereditary non-polypoid colorectal cancer, Lynch syndrome and APC-associated polyposis conditions [1].

A multicentre analysis from Victoria, Australia examining 3458 patients who underwent CTC over a 5-year period demonstrated a perforation rate of 0.06% (two cases) [5]. Both of these cases had recently undergone colonoscopy, and were managed conservatively. Most described cases of perforation following CTC occur in the rectum or sigmoid colon, and have identifiable causes such as over inflation of the rectum, obstructing malignancy, active inflammation or anastomotic stricture [6].

Perforation from CTC is a rare complication, seldom requiring surgical intervention, with low morbidity likely due to rapid diagnosis in patients with a bowel-prepped colon. This report highlights the need for high suspicion for CTC complications, despite their rarity, and the utility of diagnostic laparoscopy for diagnosis in atypical presentations.

## CONFLICT OF INTEREST STATEMENT

None declared.

## FUNDING

None.
